# TET2 and MEG3 promoter methylation is associated with acute myeloid leukemia in a Hainan population

**DOI:** 10.18632/oncotarget.15440

**Published:** 2017-02-17

**Authors:** Hongxia Yao, Mengling Duan, Lie Lin, Congming Wu, Xiangjun Fu, Hua Wang, Li Guo, Wenting Chen, Li Huang, Dan Liu, Ruo Rao, Shuwen Wang, Yipeng Ding

**Affiliations:** ^1^ Department of Hematology, Hainan General Hospital, Haikou, Hainan, 570311, P.R. China; ^2^ Department of Emergency, Hainan General Hospital, Haikou, Hainan, 570311, P.R. China

**Keywords:** AML, Hainan, LncRNA MEG3, TET2, rtPCR

## Abstract

The promoter of *MEG3*, which encodes the long non-coding RNA (lncRNA) MEG3, is often hypermethylated in acute myeloid leukemia (AML). Additionally, the Tet methylcytosine dioxygenase 2 gene (*TET2*) is frequently inactivated, which can lead to impaired DNA methylation and promote AML development. We examined the association between *TET2* and *MEG3* promoter hypermethylation in Hainan patients with AML. The expression of MEG3, TET2, miR-22-3p, and miR-22-5p was assessed in bone marrow samples from AML patients and healthy controls using real-time quantitative PCR. Using Sequenom MassARRAY technology, we compared *MEG3* promoter methylation in AML patients and healthy controls. MEG3 expression was lower in AML patients than in the controls (P = 0.136). Moreover, there was greater methylation of *MEG3* promoter in the AML patients than the controls (P < 0.05). Methylation of the *MEG3* promoter correlated negatively with *TET2* expression (P < 0.05, r < 0). Likewise there was a negative correlation between TET2 activity and *MEG3* promoter methylation (P < 0.05, r < 0). These results suggest that hypermethylation of the *MEG3* promoter in AML may result from decreased TET2 activity. These data provide insight into the molecular mechanisms underlying AML development and progression.

## INTRODUCTION

Acute myeloid leukemia (AML) is a frequently fatal malignant disease of hematopoietic stem and progenitor cells. The molecular and phenotypic characteristics of AML are highly heterogeneous [1]. It can arise from a series of genetic and epigenetic alterations that disrupt the differentiation, proliferation, and survival of myeloid progenitor cells [2]. The incidence of AML peaks in early childhood and late adulthood [3]. Although the survival rate among younger AML patients has improved, the prognosis of older patients is still poor.

Long non-coding RNAs (lncRNAs) are a heterogeneous class of RNAs greater than 200 nucleotides in length [4]. Many studies have indicated lncRNAs are important for cell cycle control [5], survival [6], migration [7], and metabolism [8]. LncRNAs participate in multiple networks that control cellular differentiation and development [9], and alterations in lncRNA expression/regulation have been associated with many diseases including cancer [10]. Recently, more and more studies have shown that lncRNAs are deregulated in a wide variety of cancers [11, 12]. Several studies have assessed the roles of lncRNAs such as ANRIL, lncRNA-P21, MEG3, Dleu2, HOTAIRM1, EGO, and lncRNA-a7 in leukemia. The results highlight the importance of investigating lncRNAs as diagnostic, prognostic, and therapeutic targets [13]. The maternally expressed gene 3 (*MEG3*) gene, located on chromosome 14q32, encodes a myelocyte-related lncRNA that has been implicated in several human malignancies [14]. However, the function of MEG3 has not been elucidated [15]. MEG3 is involved in both physiological and pathological processes. For example, it participates in signaling cascades involved in cell proliferation and differentiation, survival, and angiogenesis. Dysregulation of MEG3 has been associated with several types of cancer [16]. Previous studies have indicated that loss of MEG3 expression in cancer can result from hypermethylation of the *MEG3* promoter as well as the intergenic germline-derived differentially methylated region [16–18]. Intriguingly, hypermethylation of the *MEG3* promoter has been observed in approximately 50% of patients with myelodysplastic syndrome (MDS) and AML [19]. These results were confirmed in an independent analysis of 40 AML patient samples [20]. Hypermethylation of the *MEG3* promoter was correlated with decreased overall survival and is a prognostic marker in myeloid malignancies [19]. Thus, aberrant methylation of the *MEG3* promoter may promote AML progression [19, 21]. However, the mechanisms underlying hypermethylation of the *MEG3* promoter in AML are unclear.

Tet methylcytosine dioxygenase 2 (*TET2*) is a putative tumor suppressor gene located on chromosome 4q24.1 [22]. Mutations in *TET2* have been observed in a variety of myeloid disorders [23]. Subsequent sequencing analysis revealed that *TET2* mutations are present in approximately 7%−23% of AML patients [24–26] and in 14%−55% of patients with other myeloid malignancies [23, 24, 27]. Reduced TET2 activity and 5-hydroxymethylcytosine (5hmC) levels were observed in AML, MDS, chronic myelomonocytic leukemia (CMML), lymphoid leukemia, and other patients with hematological malignancies. Thus, *TET2* inactivation and *MEG3* promoter methylation frequently coexist.

Micro RNAs (miRNAs) regulate many cellular processes including cell proliferation, differentiation, development, apoptosis, metabolism, and hematopoiesis [28]. Interestingly, miRNA 22 (miR-22) negatively regulates TET2 expression, which results in a decrease in 5hmC and an increase in the methylation of the promoters of multiple genes. Here, we investigated the relationship between *TET2* inactivation and *MEG3* promoter methylation in Hainan patients with AML.

## RESULTS

### Analysis of MEG3, TET2, miR-22-3p, and miR-22-5p expression, and *MEG3* promoter methylation

**Figure 1:**
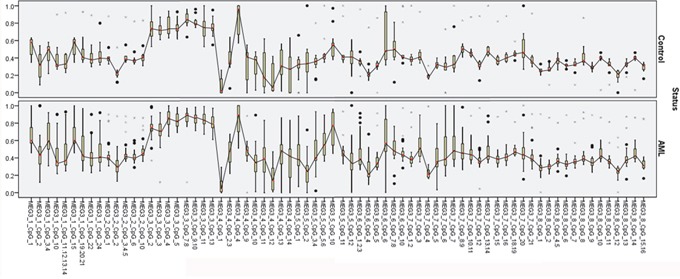
*MEG3* expression diagnosis effect analysis

In Table 1 MEG3 expression was significantly reduced in the AML compared to the control group. TET2, miR-22-3p, and miR-22-5p expression was not significant in either group. Analysis of *MEG3* promoter methylation revealed no significant differences in 19 CpG units between the AML and control groups: MEG3_1_CpG_1, MEG3_1_CpG_3.4, MEG3_1_CpG_15, MEG3_2_CpG_2, MEG3_2_CpG_6, MEG3_2_CpG_10, MEG3_3_CpG_4, MEG3_3_CpG_5, MEG3_3_CpG_11, MEG3_4_CpG_9, MEG3_5_CpG_5.6, MEG3_5_CpG_10, MEG3_7_CpG_6, MEG3_7_CpG_7, MEG3_7_CpG_12, MEG3_8_CpG_7, MEG3_8_CpG_9, MEG3_8_CpG_11, and MEG3_8_CpG_13 (Figure 1).

**Table 1:** Analysis of MEG3, TET2, miR-22-3p, and miR-22-5p expression, and *MEG3* promoter methylation

		Mean	SD	P	OR	95% CI		P
MEG3 2-ΔΔCt	Control	2.095	3.725	0.021	1.00			
	Case	0.765	1.156		3.80	0.66	21.97	0.136
TET2 2-ΔΔCt	Control	1.259	0.751	0.214	1.00			
	Case	1.069	0.783		1.75	0.40	7.71	0.459
miR-22-3p 2-ΔΔCt	Control	3.107	6.433	0.857	1.00			
	Case	4.600	17.543		2.16	0.38	12.30	0.385
miR-22-5p 2-ΔΔCt	Control	2.064	4.495	0.857	1.00			
	Case	4.079	8.213		0.63	0.12	3.33	0.588
MEG3_1_CpG_1	Control	0.528	0.104	0.034	1.00			
	Case	0.644	0.121		7.19	0.77	66.89	0.083
MEG3_1_CpG_3.4	Control	0.442	0.120	0.003	1.00			
	Case	0.630	0.176		3.09	0.49	19.62	0.232
MEG3_1_CpG_15	Control	0.528	0.104	0.034	1.00			
	Case	0.644	0.121		7.19	0.77	66.89	0.083
MEG3_2_CpG_2	Control	0.231	0.094	0.005	1.00			
	Case	0.324	0.150		10.01	0.92	108.78	0.058
MEG3_2_CpG_6	Control	0.403	0.125	0.010	1.00			
	Case	0.430	0.111		6.12E+09	0.00	.	0.999
MEG3_2_CpG_10	Control	0.432	0.126	0.006	1.00			
	Case	0.492	0.123		43.54	2.25	843.64	0.013
MEG3_3_CpG_4	Control	0.771	0.108	0.044	1.00			
	Case	0.855	0.104		4.39	0.66	28.96	0.125
MEG3_3_CpG_5	Control	0.750	0.086	0.027	1.00			
	Case	0.825	0.090		0.04	1.21	202.945	0.035
MEG3_3_CpG_11	Control	0.755	0.137	0.031	1.00			
	Case	0.847	0.083		24.24	1.26	466.05	0.035
MEG3_4_CpG_9	Control	0.329	0.202	0.049	1.00			
	Case	0.472	0.159		5.87	0.38	91.13	0.206
MEG3_5_CpG_5.6	Control	0.468	0.173	0.024	1.00			
	Case	0.567	0.162		5.09	0.66	39.51	0.12
MEG3_5_CpG_10	Control	0.561	0.168	0.030	1.00			
	Case	0.722	0.194		2.79	0.44	17.62	0.276
MEG3_7_CpG_6	Control	0.293	0.187	0.016	1.00			
	Case	0.495	0.264		6.72	0.77	58.91	0.086
MEG3_7_CpG_7	Control	0.420	0.230	0.021	1.00			
	Case	0.538	0.194		21.99	1.20	401.45	0.037
MEG3_7_CpG_12	Control	0.357	0.176	0.072	1.00			
	Case	0.383	0.153		40.16	2.00	805.15	0.016
MEG3_8_CpG_7	Control	0.308	0.078	0.048	1.00			
	Case	0.378	0.112		22.52	1.07	473.11	0.045
MEG3_8_CpG_9	Control	0.292	0.030	0.011	1.00			
	Case	0.371	0.138		9.32	0.64	134.89	0.102
MEG3_8_CpG_11	Control	0.308	0.078	0.048	1.00			
	Case	0.378	0.112		22.52	1.07	473.11	0.045
MEG3_8_CpG_13	Control	0.315	0.057	0.008	1.00			
	Case	0.409	0.151		7.80	0.58	104.79	0.121

P < 0.05 indicates statistical significance; OR: odds ratio; 95% CI: 95% confidence interval.

### Analysis of the relationship between *MEG3* promoter methylation, and MEG3 and TET2 expression

Spearman's rank correlation coefficient analysis indicated there was no linear correlation between *MEG3* promoter methylation and MEG3 expression. However, a negative correlation between *MEG3* promoter methylation and MEG3 expression was observed in the AML group (57 methylation units) (Table 2). Analysis of the relationship between TET2 expression and *MEG3* promoter methylation revealed a positive correlation between one CpG unit (MEG3_5_CpG_5.6) and TET2 expression in the control group. A negative correlation between *MEG3* promoter methylation (8 CpG units) and TET2 expression was observed in the AML group (Table 3).

**Table 2:** Spearman's rank correlation analysis of *MEG3* promoter methylation and expression

MEG3 2-ΔΔCt	Control		Case	
	r	P	r	P
MEG3_1_CpG_1	0.236	0.460	−0.632	0.002
MEG3_1_CpG_2	0.049	0.880	−0.390	0.080
MEG3_1_CpG_3.4	0.275	0.388	−0.524	0.015
MEG3_1_CpG_10	0.014	0.965	−0.736	0.000
MEG3_1_CpG_11.12.13.14	−0.057	0.861	−0.663	0.001
MEG3_1_CpG_15	0.236	0.460	−0.632	0.002
MEG3_1_CpG_19.20.21	0.056	0.862	−0.624	0.003
MEG3_1_CpG_22	−0.021	0.948	−0.339	0.133
MEG3_1_CpG_24	−0.025	0.940	−0.647	0.002
MEG3_2_CpG_1	0.050	0.878	−0.492	0.028
MEG3_2_CpG_2	−0.158	0.623	−0.317	0.174
MEG3_2_CpG_3.4.5	−0.099	0.759	−0.516	0.020
MEG3_2_CpG_6	0.273	0.391	−0.070	0.769
MEG3_2_CpG_10	−0.145	0.653	−0.339	0.144
MEG3_3_CpG_2	0.237	0.483	−0.306	0.217
MEG3_3_CpG_3	0.032	0.926	−0.107	0.671
MEG3_3_CpG_4	−0.005	0.989	−0.149	0.555
MEG3_3_CpG_5	0.146	0.668	−0.419	0.084
MEG3_3_CpG_7.8	−0.036	0.915	−0.135	0.594
MEG3_3_CpG_9.10	−0.060	0.861	−0.020	0.938
MEG3_3_CpG_11	0.224	0.508	−0.506	0.032
MEG3_3_CpG_13	0.046	0.894	−0.548	0.019
MEG3_4_CpG_1	−0.125	0.685	−0.232	0.312
MEG3_4_CpG_2.3	−0.179	0.558	−0.439	0.046
MEG3_4_CpG_4	0.243	0.424	0.061	0.797
MEG3_4_CpG_9	−0.081	0.802	−0.672	0.006
MEG3_4_CpG_10	0.069	0.823	−0.084	0.716
MEG3_4_CpG_11	−0.144	0.656	−0.644	0.003
MEG3_4_CpG_12	−0.138	0.653	−0.369	0.100
MEG3_4_CpG_13	−0.213	0.484	−0.232	0.311
MEG3_4_CpG_14	−0.124	0.685	−0.220	0.364
MEG3_5_CpG_1	−0.072	0.815	−0.491	0.024
MEG3_5_CpG_2	0.637	0.026	−0.605	0.004
MEG3_5_CpG_3.4	−0.105	0.734	−0.535	0.012
MEG3_5_CpG_5.6	0.274	0.389	−0.664	0.001
MEG3_5_CpG_10	0.098	0.761	−0.492	0.032
MEG3_5_CpG_11	0.290	0.336	−0.661	0.001
MEG3_5_CpG_12	0.391	0.187	−0.497	0.022
MEG3_6_CpG_1.2.3	0.198	0.538	−0.564	0.008
MEG3_6_CpG_4	−0.093	0.775	−0.609	0.003
MEG3_6_CpG_5	−0.302	0.316	−0.482	0.027
MEG3_6_CpG_7.8	−0.201	0.511	−0.662	0.001
MEG3_7_CpG_3	0.274	0.415	−0.562	0.012
MEG3_7_CpG_4	−0.393	0.232	−0.592	0.008
MEG3_7_CpG_5	0.156	0.648	−0.640	0.003
MEG3_7_CpG_8.9	−0.415	0.205	−0.530	0.020
MEG3_7_CpG_15	−0.111	0.746	−0.528	0.020
MEG3_7_CpG_20	−0.187	0.582	−0.486	0.035
MEG3_8_CpG_1	0.290	0.387	−0.554	0.017
MEG3_8_CpG_4.5	0.602	0.038	−0.599	0.009
MEG3_8_CpG_7	0.392	0.207	−0.498	0.035
MEG3_8_CpG_9	0.252	0.455	−0.734	0.001
MEG3_8_CpG_10	−0.125	0.699	−0.503	0.033
MEG3_8_CpG_11	0.392	0.207	−0.498	0.035
MEG3_8_CpG_13	0.004	0.991	−0.610	0.007
MEG3_8_CpG_14	−0.125	0.699	−0.503	0.033
MEG3_8_CpG_15.16	0.077	0.811	−0.476	0.046

P < 0.05 indicates statistical significance.

**Table 3:** Spearman's rank correlation analysis of *MEG3* promoter methylation and TET2 expression

TET2 2-ΔΔCt	Control		Case	
	r	P	r	P
MEG3_1_CpG_3.4	0.479	0.115	−0.414	0.049
MEG3_1_CpG_10	0.319	0.312	−0.459	0.028
MEG3_1_CpG_11.12.13.14	0.371	0.235	−0.435	0.038
MEG3_3_CpG_5	0.511	0.108	−0.484	0.031
MEG3_4_CpG_11	0.227	0.502	−0.448	0.042
MEG3_5_CpG_5.6	0.596	0.041	−0.458	0.032
MEG3_5_CpG_11	0.392	0.208	−0.437	0.042
MEG3_7_CpG_5	−0.297	0.374	−0.486	0.025

P < 0.05 indicates statistical significance.

We performed multivariable linear regression analysis of the relationship between *MEG3* promoter methylation and MEG3 expression in Table 4. After adjusting for sex and age, we identified as association between *MEG3* promoter methylation (7 CpG units) and MEG3 expression (P < 0.05). Among the CpG units, linear changes in MEG3 expression were correlated with MEG3_4_CpG_9 (control, B = −21.60, P = 0.01; case, B = −10.56, P < 0.001) and MEG3_5_CpG_2 (control, B = 20.50, P < 0.001; case, B = −6.19, P = 0.02). In Table 5 we also found that six CpG methylation units were correlated with TET2 expression (P < 0.05). There was no significant correlation in the control group but an inverse linear correlation was observed in the case group (B < 0).

**Table 4:** Multivariable linear regression analysis of *MEG3* promoter methylation and expression

MEG3 2-ΔΔCt		B	P	95% CI	
MEG3_1_CpG_1	Control	3.74	0.53	−6.40	11.49
	Case	−8.68	0.09	−19.17	1.81
MEG3_1_CpG_3.4	Control	3.74	0.30	−3.96	11.44
	Case	−6.62	0.00	−10.28	−2.97
MEG3_4_CpG_9	Control	−21.60	0.01	−37.04	−6.16
	Case	−10.56	0.00	−13.54	−7.57
MEG3_5_CpG_2	Control	20.50	0.00	12.17	28.82
	Case	−6.19	0.02	−11.34	−1.05
MEG3_5_CpG_12	Control	11.27	0.18	−6.43	28.97
	Case	−3.97	0.05	−7.91	−0.03
MEG3_8_CpG_4.5	Control	6.92	0.20	−4.47	18.31
	Case	−18.80	0.04	−36.47	−1.14
MEG3_8_CpG_9	Control	13.78	0.15	−6.28	33.83
	Case	−12.68	0.02	−23.05	−2.31

P < 0.05 indicates statistical significance; 95% CI: 95% confidence interval.

**Table 5:** Multivariable linear regression analysis of *MEG3* promoter methylation and TET2 expression

TET2 2-ΔΔCt		B	P	95% CI	
MEG3_1_CpG_10	Control	0.040	0.593	−0.125	0.204
	Case	−0.152	0.040	−0.296	−0.008
MEG3_1_CpG_24	Control	0.024	0.741	−0.136	0.184
	Case	−0.119	0.047	−0.236	−0.002
MEG3_2_CpG_2	Control	0.059	0.104	−0.015	0.134
	Case	−0.091	0.028	−0.171	−0.012
MEG3_5_CpG_11	Control	0.024	0.351	−0.032	0.081
	Case	−0.057	0.001	−0.084	−0.03
MEG3_6_CpG_1.2.3	Control	−0.023	0.213	−0.095	0.049
	Case	−0.155	0.048	−0.307	−0.002
MEG3_8_CpG_2	Control	−0.038	0.509	−0.166	0.089
	Case	−0.026	0.020	−0.047	−0.005

P < 0.05 indicates statistical significance; 95% CI: 95% confidence interval.

### Analysis of the correlation between TET2 expression and miR-22-3p, miR-22-5p, and MEG3 expression

We did not observe a correlation between miR-22-3p, miR-22-5p, and TET2 expression in either the AML or control group before or after adjustment for age and gender (Table 6). We did observe a positive correlation between TET2 and MEG3 expression in the AML group (Spearman's rank correlation coefficient, r = 0.634 > 0). However, no significant correlation was detected after adjustment for age and gender. Finally, multivariable linear regression analysis indicated TET2 expression was positively correlated with MEG3 expression in the control group (B = 0.708 > 0).

**Table 6:** Analysis of the correlation between TET2 expression, and miR-22-3p and miR-22-5p expression

TET2 2-ΔΔCt	Control		Case	
	r	P	r	P
miR-22-3p 2-ΔΔCt	0.341	0.181	0.202	0.334
miR-22-5p 2-ΔΔCt	0.118	0.653	0.072	0.731

P < 0.05 indicates statistical significance.

### Analysis of MEG3 expression as a diagnostic test

**Figure 2:**
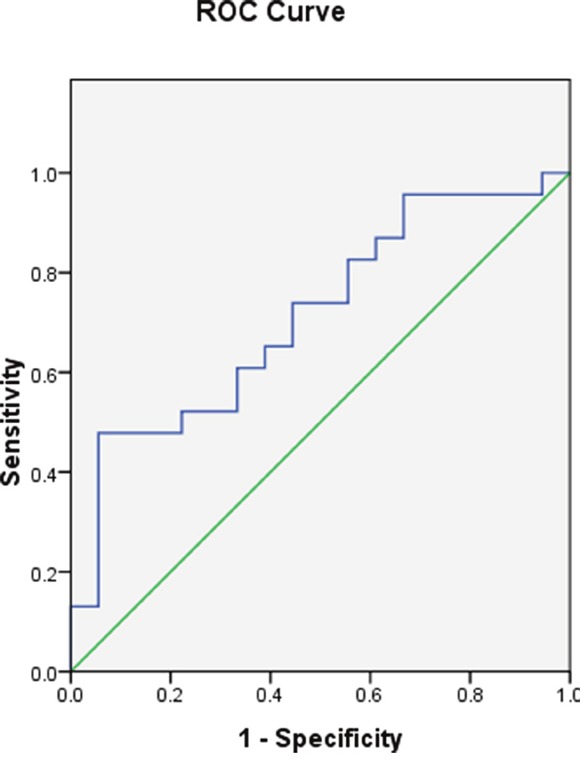
*MEG3* promoter methylation in the AML and control groups

ROC curve analysis showed MEG3 expression was effective as a diagnostic (area under the curve = 0.713, 95% confidence interval [CI] = 0.554−0.871, P = 0.021) (Figure 2).

## DISCUSSION

Aberrant promoter methylation can result in silencing of gene expression and contribute to the development of leukemia. Changes in DNA methylation state (particularly hypermethylation of tumor suppressor genes) is a diagnostic and prognostic marker in patients with hematological malignancies [29]. Previous studies of the role of DNA methylation in AML have achieved conflicting results. Analysis of epigenetic patterns in AML could enable identification of new patient subgroups and/or provide new prognostic biomarkers. Here, we assessed the relationship between *MEG3* promoter methylation and MEG3, TET2, miR-22-3p, and miR-22-5p expression.

*MEG3* is a maternally expressed gene on that encodes a lncRNA with a length of 1.6 kb [30, 31]. The functions of MEG3 have not yet been defined. However, it has been implicated in normal physiological processes as well as tumorigenesis [32]. *MEG3* promoter methylation was also correlated with reduced overall survival, and could serve as a prognostic marker in myeloid malignancies [15]. Promoter methylation is not always disease-related. It also occurs under normal conditions and is important for the expression of growth factors and their receptors, cytokines, and various other molecules during normal myeloid development [21]. Promoter hypermethylation and aberrant silencing of genes involved in cell adhesion, cell cycle regulation, and tumor suppression has been observed in hematological malignancies such as MDS and AML. These alterations are thought to occurs at approximately the same frequency as mutations [17].

Reduced MEG3 expression has been observed in tumor tissue. For example, MEG3 expression was significantly lower in non-functional pituitary adenoma compared to normal tissue [14, 33]. Reduced MEG3 expression has also been observed in breast, cervical, colon, liver, lung, and prostate cancer cell lines [14, 34]. We observed reduced MEG3 expression in the AML compared to the control group. Because MEG3 enhances the activity of the tumor suppressor P53, down-regulation of MEG3 expression may promote cancer progression. Indeed, down-regulation of MEG3 expression has been observed in approximately 50% of AML patients and is mediated by promoter hypermethylation.

Altered DNA methylation is an important mechanism underlying tumor development and progression [35, 36]. TET2 catalyzes the oxidation of 5-methylcytosine (5mC) to 5hmC, and decreased TET2 activity can result in an altered methylation pattern (e.g. promoter hypermethylation) [37]. *TET2* inactivation can cause impaired DNA demethylation and ultimately promote AML development. TET2 inactivation promotes hematological malignancies [38]. The TET enzyme catalyzes the oxidation of 5mC to 5hmC, resulting in active DNA demethylation. The TET family of proteins includes TET1, TET2, and TET3. *TET2* inactivation is the most common alteration in hematological malignancies. TET2 activity and 5hmC levels were shown to be reduced in AML, MDS, CMML, lymphocytic leukemia, and other hematological malignancies [38]. Mutations in *TET2* and inactivation through methylation have been observed in AML patients, and impact the complete remission rate and disease-free survival. Thus, *TET2* inactivation may promote the development and progression of a variety of hematological malignancies including AML [39]. We observed differences in the methylation level of the *MEG3* promoter between the AML and control groups. Additionally, we observed a negative correlation between *MEG3* promoter methylation and MEG3 expression. Finally, we determined that *MEG3* promoter methylation was negatively correlated with TET2 expression.

TET2 expression is negatively regulated by miR-22, which reduces the expression of 5hmC and enhancing the methylation of multiple genes [40]. However, we did not detect an association between miR-22 and TET2 expression in AML. Our data demonstrate inactivation of *TET2* and hypermethylation of the *MEG3* promoter in AML. We hypothesize that *TET2* inactivation causes hypermethylation of the *MEG2* promoter based on the negative correlation between TET2 expression and *MEG3* promoter methylation. TET2 expression and MEG3 promoter hypermethylation may serve as prognostic markers in AML and lead to new targeted therapeutics.

## MATERIALS AND METHODS

### Patients and samples

Bone marrow samples were obtained from 29 patients with AML (diagnosed according to the French-American-British criteria [41]) who were treated at the People's Hospital of Hainan Province between February 2014 and August 2015. The control population consisted of 20 healthy volunteers. The protocol was approved by the People's Hospital of Hainan Province. Written informed consent was obtained from all patients.

### Quantitative real-time PCR

Total RNA was extracted from frozen tissue samples or cells using the TRIzol reagent (Invitrogen, Carlsbad, CA, USA) according to the manufacturer's protocol. A total of 1 μg of RNA was reverse transcribed using the TIANScript RT Kit (TIANGEN, Beijing, China). Quantitative real-time PCR was performed using the BIO-RAD iQ5 Real-Time System (BIO-RAD, Hercules, CA, USA) and SYBR Green (TIANGEN) as a double-stranded DNA-specific dye. We performed the cDNA synthesis using a Thermo Script RT-qPCR System (Invitrogen). Target genes were amplified with primers designed using the Primer Premier Version 5.0 software. The following protocol was used for real-time PCR: 95°C for 2 min followed by 40 cycles at 95°C for 15 sec, and then 60°C for 1 min. Standard curves were generated for each assay to produce a linear plot of threshold cycle (Ct) against log (dilution). Target gene expression was quantified using the standard curve method. Data are presented as relative Ct values (n = 6). MEG3 and TET2 expression was normalized to GAPDH, while miR-22-3p and miR-22-5p levels were normalized to U6 snRNA. The relative levels of MEG3, TET2, miR-22-3p, and miR-22-5p were calculated using the 2-ΔΔCt method [(Ct, HOTAIR - Ct, GAPDH, U6) - (Ct, HOTAIR - Ct, GAPDH, U6) control].

### DNA extraction and bisulfite modification

DNA was extracted from bone marrow tissue collected into EDTA-containing tubes using a Qiagen DNA Extraction kit. Bisulfite treatment was performed using the EZ DNA Methylation kit (Zymo Research, Irvine, CA, USA) and the manufacturer's protocol. Quantification of DNA methylation was performed using the Sequenom MassARRAY platform and the EpiTYPER software (Sequenom, San Diego, CA, USA). This platform contained 125 CpG sites. There were 8 CpG units that resulted from cleavage after T, and each unit included single or multiple CpG sites. Using the Mass Cleave assay (Sequenom), we quantitatively assessed the levels of DNA methylation at single CpG units consisting of at least one CpG dinucleotide. Sequenom MassARRAY primers were designed to cover all possible alternative CpG cleavage sites using the Methyl Primer Express software v1.0. Amplicons were designed using the Sequenom Epityper software v.1.0. The PCR conditions were the following: 94°C for 5 min, 94°C for 30 s, 64.6°C for 30 s (annealing), 72°C for 1 min (elongation), and 72°C for 7 min.

### Statistical analysis

Statistical analyses were performed using the SPSS 17.0 software (SPSS, Chicago, IL, USA). Demographic and clinical data are reported as the mean, median, or a proportion. The data were analyzed using Student's t-tests or one-way analysis of variance, and a P value < 0.05 was considered statistically significant. Mann-Whitney U tests were performed using the GraphPad Prism 5 software. Spearman's correlation was used to assess differences in methylation levels. Finally, receiver operating characteristic (ROC) curves were used to evaluate *MEG3* promoter methylation as a diagnostic marker for AML.
